# Supplementary material for: Diversity of cyanobacteria from thermal muds (Balaruc-Les-Bains, France) with the description of Pseudochroococcus coutei gen. nov., sp. nov
Source: FEMS Microbes. 2021 Apr 22;2:xtab006. doi: 10.1093/femsmc/xtab006 (PMC10117791; doi:10.1093/femsmc/xtab006)
Supplement: xtab006_Supplemental_Files [file xtab006_supplemental_files.zip › Supplementary materials_Revision.docx]

**Supplementary materials:**

**
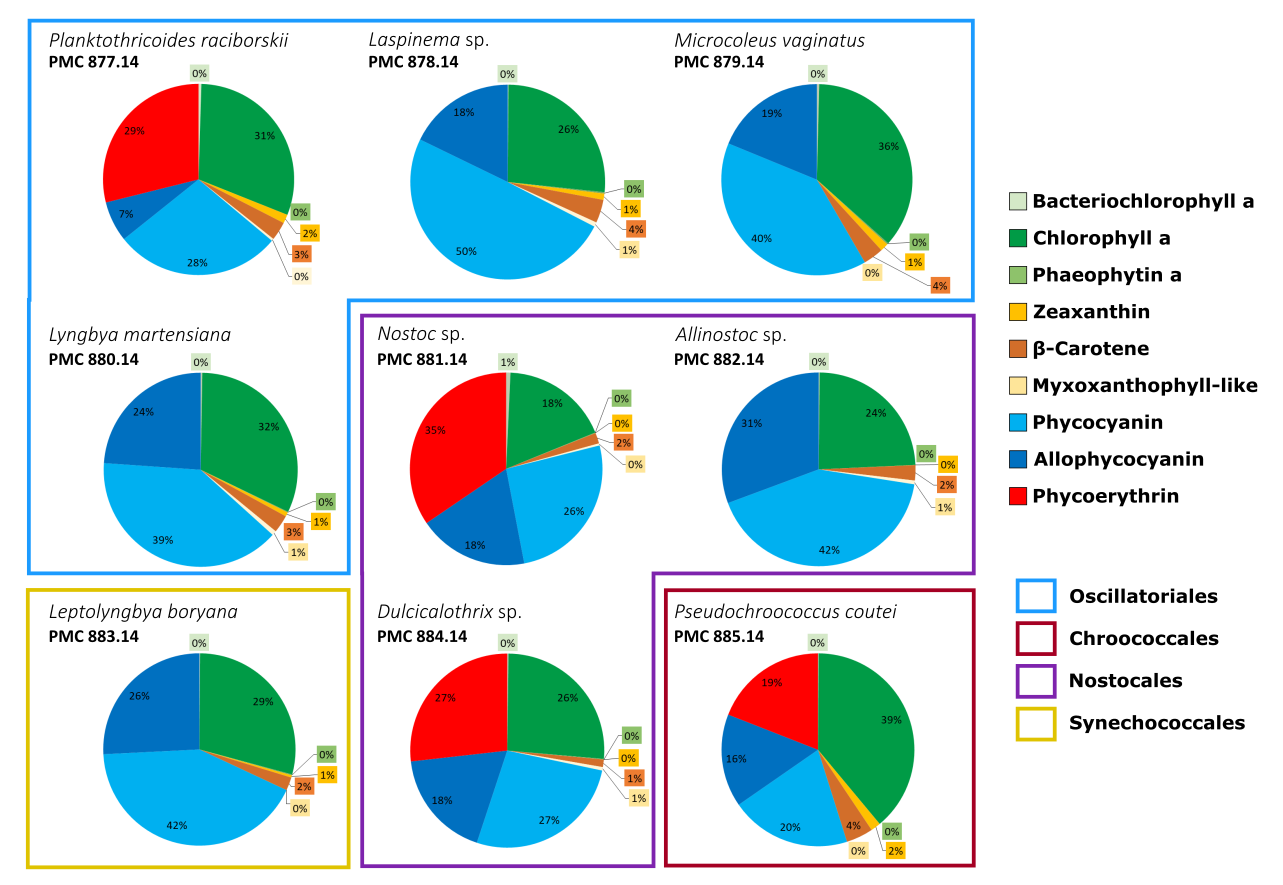
**

**Figure S1.** Pigments composition (%) of the nine studied strains, isolated from Thermes de Balaruc-Les-Bains. PMC: Paris Museum Collection, Museum National of Natural History.

*Nostoc flagelliforme* CCNUN1 (NZ CP0247851:81819-83309)

*Nostoc punctiforme* PCC 73102

*Nostoc* sp. PCC 9709 (AF027654.1)

*Nostoc calcicola* III

*Nostoc* sp. ATCC 53789 (AF062638.1)

*Nostoc edaphicum* X

***Nostoc* sp. PMC 881.14 (MT984289)**

*Nostoc ellipsosporum* V

*Nostoc muscorum* Lukesova 1/87 (AM711523.1)

*Desmonostoc muscorum* I (AJ630451.1)

*Desmonostoc* sp. PCC 7422 (HG004586.1)

*Desmonostoc* sp. PCC 9230 (HG004585.1)

*Nostoc linckia* NIES-25 (NZ AP0182221:c3653887-3652392)

*Trichormus variabilis* ATCC 29413 (HF678501.1)

*Nostoc* sp. PCC 7120

*Nostoc* sp. PCC 7524

*Trichormus doliolum* 1

*Nostoc* sp. PCC 9426 (AM711538.1)

*Trichormus azollae* Kom-BAI/1983

*Nostoc* sp. PCC 6720 (DQ185240.1)

*Nostoc* sp. PCC 7107

*Minunostoc cylindricum* CHAB 5844-1 (MK045295.1)

*Minunostoc cylindricum* CHAB 5843 clone 3 (MK045297.1)

*Aliinostoc tiwarii* LI PS (MH497064.1)

*Aliinostoc* *constrictum* SA30 (MK503793.1)

***Aliinostoc* sp. PMC 882.14 (MT984291)**

*Aliinostoc soli* ZH1(3) PS (MH497065.1)

*Aliinostoc magnkinetifex* SA18 (MK503791.1)

*Aliinostoc catenatum* SA24 (MK503792.1)

*Aliinostoc morphoplasticum* NOS (KY403996.1)

*Aliinostoc* sp. CENA548 (KX458492.1)

*Anabaena* sp. 90

*Anabaena tenericaulis* 08 10

*Dolichospermum solitarium* PMC 196.03

*Dolichospermum flos-aquae* PMC 207.03

*Cuspidothrix issatschenkoi* 0tu37s7

*Trichormus variabilis* HINDAK 2001/4 (AJ630456.1)

*Trichormus variabilis* GREIFSWALD (AJ630457.1)

*Anabaena cylindrica* PCC 7122

*Anabaena bergii* PMC 314.07

*Nodularia* sp. PCC 73104

*Nodularia spumigena* CCY9414

*Calothrix* sp. PCC 6303

*Calothrix* sp. PCC 8909 (AM230693.1)

*Calothrix* sp. XP11C (AM230698.1)

***Dulcicalothrix* sp. PMC 884.14 (MT984290)**

*Dulcicalothrix thermalis* PCC 7715 (AM230701.1)

*Dulcicalothrix desertica* PCC 7102 (NR 114995.1)

*Dulcicalothrix* sp. PCC 7103 (AM230700.1)

*Dulcicalothrix parietina* CCAP 1410/10 (HF678479.1)

*Leptolyngbya boryanum* (X84810.1)

*Leptolyngbya boryana* PCC 6306 (EF429290.1)

*Leptolyngbya boryana* IAM M-101 (AB245143.1)

*Leptolyngbya boryana* NIES-2135 (LC215287.1)

***Leptolyngbya boryana* PMC 883.14 (MT984284)**

*Leptolyngbya crispata* SEV4-3-C6 (AY239598.1)

*Leptolyngbya* sp. SEV5-3-C2 (AY239592.1)

*Leptolyngbya* sp. SEV5-3-C28 (AY239595.1)

*Leptolyngbya antarctica* ANT.L67.1 (AY493572.1)

*Leptolyngbya antarctica* ANT.LWA.1 (AY493604.1)

*Stenomitos* sp. PMC 304.07 (GQ859652.1)

*Stenomitos* sp. Ru-0-2 (MH688849.1)

*Stenomitos rutilans* HA7619-LM2 (KF417430.1)

*Leptolyngbya frigida* ANT.L53B.2 (AY493576.1)

*Leptolyngbya nostocorum* UAM 387 (JQ070063.1)

*Leptolyngbya* sp. Zehnder 1965/U140 (HM018692.1)

*Leptolyngbya mycoidea* LEGE 06108 (HQ832942.1)

*Leptolyngbya valderiana* SABC022801 (KY807918.1)

*Limnothrix redekei* 165c (AJ505943.1)

*Limnothrix redekei* PMC 272.06 (GQ859647.1)

*Nodosilinea* sp. CENA322 (KT731143.1)

*Nodosilinea nodulosa* UTEX 2910 (KF307598.1)

*Nodosilinea* sp. PMC 302.07 (GQ859653.1)

*Nodosilinea* sp. CENA523 (KF246490.1)

*Synechocystis* sp. PCC 6702 (AB041936.1)

*Synechocystis* sp. PCC 6803 (AY224195.1)

*Cyanothece* sp. ATCC 51142 (AF132771.1)

*Gloeocapsa* sp. KO30D1 (AB067579.1)

*Cyanothece* sp. WH 8902 (AY620238.1)

*Gloeothece* sp. KO11DG (AB067577.1)

*Gloeothece* sp. SK40 (AB067576.1)

*Cyanothece* sp. PCC 8801 (AF296873.1)

Cyanobacteria sp. YW-2019b strain TP201716.4 clone 06 (MN215485.1)

Cyanobacteria sp. YW-2019b strain TP201716.1 clone 03 (MN215477.1)

***Pseudochroococcus coutei* PMC 885.14 (MT985488)**

*Inacoccus carmineus* CCIBt3475 (MF072349.1)

*Inacoccus carmineus* CCIBt3418 (MF072348.1)

*Gloeocapsa* sp. PCC 73106 (AB039000.1)

Uncultured *Limnococcus* sp. Alla11otu7-1 16 (KP676759.1)

*Limnococcus limneticus* Svet06 (GQ375048.1)

*Cryptococcum komarkovaum* CCALA 054 (MF072346.1)

*Cryptococcum brasiliense* CCIBt3410 (MF072345.1)

*Chroococcus minutus* SAG 41.79 (KM019988.1)

*Chroococcus minutus* CCALA 055 (GQ375047.1)

*Chroococcus turgidus* CCIBt3508 (MF072352.1)

*Chroococcus* cf. *westii* CCALA 702 (GQ375044.1)

*Chroococcus subviolaceus* CCIBt3549 (MF072353.1)

*Chroococcus subviolaceus* CCIBt3506 (MF072351.1)

*Phormidium* cf. *nigrum* CCALA 147 (AM398779.1)

*Phormidium* cf. *irriguum* CCALA 759 (EU196638.1)

*Lyngbya martensiana* H3b/7 (JN854142.1)

***Lyngbya martensiana* PMC 880.14 (MT984286)**

*Trichodesmium thiebautii* (AF091321.1)

*Trichodesmium hildebrandtii* (AF091322.1)

*Trichodesmium erythraeum* IMS101 (113473942:3137158-3138645)

*Kamptonema animale* INDIA92 (KP221930.1)

*Kamptonema animale* CCALA 771 (KP221932.1)

*Microcoleus vaginatus* Luznice (KC633959.1)

*Microcoleus autumnalis* Arct-Ph5 (DQ493873.2)

*Microcoleus autumnalis* SAG 35.90 (EF654081.1)

*Microcoleus vaginatus* CCALA 154 (JN230342.1)

*Microcoleus vaginatus* PCC 9802 (AF284803.1)

*Microcoleus vaginatus* SAG 2502 (MK953007.1)

***Microcoleus vaginatus* PMC 879.14 (MT984285)**

*Microcoleus vaginatus* JR12 (JN230335.1)

*Microcoleus vaginatus* JR6 (JN230339.1)

*Lyngbya robusta* CCALA 966 (JN854138.1)

*Lyngbya aestuarii* PCC 7419

*Planktothricoides raciborskii* NSLA4 (AB045963.1)

***Planktothricoides raciborskii* PMC 877.14 (MT984287)**

*Planktothricoides raciborskii* NIES-207 (AB045960.1)

*Planktothricoides raciborskii* OR1-1 (B045964.1)

*Laspinema lumbricale* UTCC 476 (AF218375.1)

*Laspinema thermale* HK S5 clone cl2 (MF360989.1)

*Laspinema thermale* HK S3 clone cl2 (MF360982.1)

***Laspinema* sp. PMC 878.14 (MT984288)**

*Phormidium* cf. *terebriformis* KR2003/25 (AY575936.1)

*Laspinema etoshii* KR2008/49 (KC014068.1)

*Gloeobacter violaceus* PCC 7421 (AF132790.1)

100 / 100 / 100

100 / 100 / 100

99 / 100 / 99

86 / 95 / 79

100 / 100 / 100

95 / 90 / 63

100 / 100 / 100

52 / 62 / -

100 / 100 / 100

100 / 100 / 100

87 / 64 / 69

100 / 100 / 99

96 / 66 / 97

99 / 96 / 91

100 / 99 / 99

90 / - / 86

100 / 100 / 100

100 / 100 / 100

100 / 100 / 100

100 / 100 / 100

100 / 100 / 99

81 / 91 /68

98 / 99 / 76

87

83 / 51 / -

100 / 100 /100

100 / 100 / 100

100 / 100 / 99

100 / 100 / 100

92

100 / 100 / 100

100 / 100 / 100

100 / 100 /100

100 / 100 / 100

62 / 72 /65

100 / 100 / 100

100 /100 / 100

91 / 79 / 69

100 / 100 / 100

81 / 81 / 60

100 / 100 / 99

100 / 100 / 100

100 / 99 / 99

100 / 100 / 100

100

95 / 94 / 87

100 / 100 / 100

99 / 93 / 89

100 / 97 / 99

77 /51 / -

67 / 87 / -

94 / 95 / 96

97 / 100 / 95

83 / 93 / 84

99 / 91 / 99

62 / - / 51

50 / - / -

65 / - / -

96 /9 7 / 94

85 / 95 / -

53 / 85 / 81

99 / 98 / 99

69 / 84 / 72

99 / 93 / 93

99 / 91 / 99

100 / 99 / 99

100 / 100 / 100

55 / 53 / 84

69 / - / 67

51 / - / -

100 / - / 92

99 / 80 / 98

76 / 79 / 87

98 / 94 / 98

100 / 100 / 95

94 / 69 / 78

86 / 50 / -

53 / 95 / 95

82 / 55 / 73

59 / 69 / 59

50 / - / -

63 / 81 / 78

61 / - / -

84 / 99 / -

0.050

**Nostocales**

**Synechococcales**

**Chroochococcales**

**Oscillatoriales**

**Figure S2.** Consensus phylogenetic tree based on 16S rRNA gene sequences of representative cyanobacteria strains (129 sequences, 1446 aligned nucleotide positions, GTR+G+I model) belonging to the orders Oscillatoriales, Nostocales, Chroococcales, Synechococcales and the studied strains form Thermes de Balaruc-Les-Bains (in bold). *Gloeobacter violaceus* was used as outgroup. Numbers above branches indicate bootstrap support (>50%) from 1000 replicates. Bootstrap values are given in the following order: maximum likelihood / neighbor joining / maximum parsimony.

**Figure S3.** Consensus phylogenetic tree (Maximum likelihood tree presented) based on 16S-23S ITS gene sequences (21 sequences, 1915 aligned nucleotide positions, GTR+G+I model) of representative cyanobacteria strains belonging to the orders Chroococcales, Thermes de Balaruc-Les-Bains’s strains (in bold), other species were obtained from Genbank. *Microcystis aeruginosa* PMC 728.11 was used as an outgroup. Numbers above branches indicate bootstrap support (>50%) from 1000 replicates. Bootstrap values are given in the following order: maximum likelihood / neighbor joining / maximum parsimony.

**Figure S4.** Consensus phylogenetic tree (Maximum likelihood tree presented) based on 16S rRNA gene sequences (54 sequences, 1333 aligned nucleotide position, GTR+G+I model) of representative cyanobacteria strains belonging to the orders Synechococcales, Thermes de Balaruc-Les-Bains’s strain is indicated in bold (with brown color), other species were obtained from Genbank. *Gloeobacter violaceus* PCC 7421 was used as an outgroup. Numbers above branches indicate bootstrap support (>50%) from 1000 replicates. Bootstrap values are given in the following order: maximum likelihood / neighbor joining / maximum parsimony.

**Figure S5.** Consensus phylogenetic tree (Maximum likelihood tree presented) based on 16S rRNA gene sequences (57 sequences, 1305 aligned nucleotide positions, GTR+G+I model) of representative cyanobacteria strains belonging to the orders Oscillatoriales, Thermes de Balaruc-Les-Bains’s strains are indicated in bold (colored in blue), other species were obtained from Genbank. *Gloeobacter violaceus* PCC 7421 was used as an out-group. Numbers above branches indicate bootstrap support (>50%) from 1000 replicates. Bootstrap values are given in the following order: maximum likelihood / neighbor joining / maximum parsimony.

**Table S1.** List of GenBank accession numbers. PMC: Paris Museum Collection.

| **Order** | **Species** | **Strain number** | **16S** | **16S -23S** |
| --- | --- | --- | --- | --- |
| Chroococcales | *Pseudochroococcus coutei* | PMC 885.14 | - | MT985488 |
| Synechococcales | *Leptolyngbya boryana* | PMC 883.14 | MT984284 | - |
| Oscillatoriales | *Planktothricoides raciborskii* | PMC 877.14 | MT984287 | - |
|  | *Laspinema* sp. | PMC 878.14 | MT984288 | - |
|  | *Microcoleus vaginatus* | PMC 879.14 | MT984285 | - |
|  | *Lyngbya martensiana* | PMC 880.14 | MT984286 | - |
| Nostocales | *Nostoc* sp. | PMC 881.14 | MT984289 | - |
|  | *Aliinostoc* sp. | PMC 882.14 | MT984291 | - |
|  | *Dulcicalothrix* sp. | PMC 884.14 | MT984290 | - |

**Table S2.** Comparison of the morphological features of *Pseudochroococcus coutei* PMC 885.14 strain with other Chroococcales (*Gleocapsa, Limnococcus, Inacoccus, Cryptococcum)* genera (Fig. 4)*.* nd: not determined.

| **Taxonomic assignment** | **Strain** | **Origin** | | **Habitat** | | **Vegetative cell** | | | | | **Reference** |
| --- | --- | --- | --- | --- | --- | --- | --- | --- | --- | --- | --- |
|  |  |  |  | | Shape | | Sheath | Color | Size min.-max. (mean), µm |  | |
| *Pseudochroococcus coutei* | PMC 885.14 | France | Epilithic biofilm | | More or less spherical, oval, hemispherical after division | | Colorless, slightly lamellate | Pale green, blue-green  or grey | 14-17.8 (15.6) | This study | |
| Cyanobacteria sp. YW-2019b | TP201716.1 clone 03 (MN215477.1) | China | nd | | nd | | nd | nd | nd | Institute of Hydrobiology site web | |
| *Gleocapsa* sp. | PCC 73106 (AB039000.1) | Switzerland | Sphagnum bog | | nd | | nd | nd | nd | PCC site web collection | |
| *Limnococcus limneticus* | Svet06 (GQ375048.1) | Czech Rep | Fishpond Svet plankton | | nd | | nd | nd | 7-11 | Komărkovă et al. 2010 | |
| *Inacoccus carmineus* sp. nov. | CCIBt3411 (MF072347.1) | Brazil (Santa Virgínia Park) | Terrestrial, on concrete. | | Hemispherical to rounded | | Hyaline to intense red-colored smooth or rarely lamellate | Purple brownish  to green-brownish | 4.7–11.9 (6.7) | Gama et al. 2019 | |
| *Inacoccus carmineus* sp. nov. | CCIBt3475 (MF072349.1) | Brazil (Ilha do Cardoso State Park) | Wet Rock | | Hemispherical to rounded | | Hyaline to intense red-colored smooth or rarely lamellate | Purple brownish  to green-brownish | 4.7–11.9 (6.7) | Gama et al. 2019 | |
| *Cryptococcum komarkovaum* | CCALA 054 (MF072346.1) | Unknown | Aquatic, thermal spring | | Hemispherical to rounded solitary or rarely grouped  in few-celled colonies | | Hyaline, smooth or rarely lamellate | Blue-green to yellowish-green | 8.7–13.9 (10.8) | Gama et al. 2019 | |
| *Cryptococcum brasiliense* | CCIBt3410 (MF072345.1) | Brazil (Ilha do Cardoso State Park) | Terrestrial, on dry soil among pebbles | | Hemispherical to rounded solitary or rarely grouped in few-celled colonies | | Hyaline, smooth or rarely lamellate | Green to blue-green | 8.3–15.5 | Gama et al. 2019 | |

**Table S3.** Comparison of the morphological features of the *Leptolyngbya boryana* sp*.* PMC 883.14 strain with other *Leptolyngbya* species belonging to the same phylogenetical cluster (Fig. 8). nd: not determined.

| **Taxonomic assignment** | **Strain** | **Origin** | **Habitat** | **Trichome** | | | | | | **Vegetative cell** | | | | | | | **Reference** | |  |
| --- | --- | --- | --- | --- | --- | --- | --- | --- | --- | --- | --- | --- | --- | --- | --- | --- | --- | --- | --- |
|  |  |  |  | Color | Sheath | Shape | Shape of terminal cell | Width min.-max. (mean) µm | | | Shape | | Width min.-max. (mean), µm | | Length min.-max. (mean), µm | | |  | |
| *Leptolyngbya boryana* | PMC 883.14 | France | Freshwater, benthic, biofilms | Pale to bright blue-green | Thin, colourless, attached to the trichome | Straight, curved, distinctly constricted, | Rounded hemispherical | | 1.5-2.4 | | | Moniliform, shorter than wide | | 1.5-2.4 (2.1 ±0.24) length:width ratio = 0.75 | | 0.9 - 2 (1.52 ±0.27) | | This study | |
| *Leptolyngbya boryana* | PCC 6306 (EF429290.1) | nd | Freshwater, metaphytic, lentic | Blue-green | Absent | Long, straight or wavy, distinctly constricted, false branching | Rounded | | nd | | | More or less isodiametric | | length:width ratio = 0.78 | | length:width ratio = 0.78 | | Chakraborty et al. 2019 | |
| *Leptolyngbya boryana* | UAM 391 (JQ070062.1) | Spain Guadarrama river | Freshwater, benthic, biofilms | Intense blue-green or pale color | Hyaline, homogeneous sometimes thick, separated from the filament | Strongly constricted, false branching | Rounded | | nd | | | nd | | 2.1–3.2 | | 1.2–3.6 | | Loza et a. 2013 | |
| *Leptolyngbya boryana* | IAM M-101 (AB245143.1) | Japan | Freshwater | nd | nd | nd | nd | | nd | | | nd | | nd | | nd | | nd | |
| *Leptolyngbya boryana* | NIES-2135 (LC215287.1) | Japan | Freshwater | nd | nd | nd | nd | | nd | | | nd | | nd | | nd | | nd | |
| *Leptolyngbya foveolarum* | 1964/112 (X84808.1) | nd | nd | nd | Presence of a sheath | Constrictions at the cross-walls | nd | | nd | | | nd | | 3.8 | | 3.8 | | Nelissen et al. 1996 | |
| *Leptolyngbya boryanum* | PCC 73110 (X84810.1) | nd | Freshwater | nd | Variable | Constrictions at the cross-walls | nd | | nd | | | nd | | 1.9 – 2.6 | | 1.9 – 2.9 | | Nelissen et al. 1996 | |

**Table S4.** Comparison of the morphological features of the *Planktothricoides raciborskii* PMC 877.14 strain with other *Planktothricoides* species belonging to the same phylogenetical cluster (Fig. 9). nd: not determined.

| **Taxonomic assignment** | **Strain** | **Origin** | **Habitat** | **Trichome** | | | | | **Vegetative cell** | | | | **Reference** | |
| --- | --- | --- | --- | --- | --- | --- | --- | --- | --- | --- | --- | --- | --- | --- |
|  |  |  |  | Color | Sheat | Shape | Shape of terminal cell | Width min.-max. (mean) µm | | Shape | Width min.-max. (mean), µm | Length min.-max. (mean), µm | |  |
| *Planktothricoides raciborskii* | PMC 877.14 | France | Freshwater, mud | Pale-green, brown | Thin, colourless | Straight +/- curved | Rounded conical | 5-7.3 (2020) | | Cylindrical, shorter than wide | 5-7.3 (6 ±0.56) | 3.3-5.4 (4.48 ±0.46) | | This study |
| Candidatus *Planktothricoides rosea* | OP (KC407688.1) | Guadeloupe | Marine mangrove Mat on thesediment from the periphyton | Pink | Thin, clear | Straight slightly waved | Slightly rounded  without calyptra | 5–10 | | Disk-shaped greater in width than in length | 5–10 | 1.25-2.5 | | Guidi-Rontani et al. 2014 |
| Candidatus *Planktothricoides niger* | OB (KC407687.1) | Guadeloupe | Marine mangrove Mat on the sediment from the periphyton | Black | Thin, clear | Straight slightly waved | Slightly rounded  without calyptra | 5–10 | | Disk-shaped greater in width than in length | 5–10 | 1.25-2.5 | | Guidi-Rontani et al. 2014 |
| *Planktothricoides raciborskii* | OR1-1 (B045964.1) | Thailand  Bangkok | Freshwater planktic in lakes | Pale blue-green yellow-green | nd | Straight attenuated at the ends | Rounded without calyptra | nd | | nd | 5.4–2.2 (Group IV) : 5.4–12.2 | (Group IV) : 1.7–6.7   (3.3 ± 0.6) | | Suda et al. 2002 |
| *Planktothricoides raciborskii* | NSLA4 (AB045963.1) | Australia New South Wales Lake Alexandrina | Freshwater planktic in lakes | nd | nd | Straight attenuated at the ends | nd | nd | | nd | (Group IV) 5.4–12.2 | (Group IV) : 1.7–6.7 | | Suda et al. 2002 |
| *Planktothricoides raciborskii* | NIES-207 (AB045960.1) | Japan Ibaraki Lake Kasumigaura | Freshwater planktic in lakes | Pale blue-green yellow-green | nd | Straight attenuated at the ends | Tapered, bent | nd | | nd | 5.4–9.8 (Group IV) 5.4–12.2 | (Group IV) : 1.7 –6.7 | | Suda et al. 2002 |
| *Planktothricoides* sp. | SR001 (NZ_LIUQ01000113.1) | Singapore | Freshwater, planktic reservoir | nd | nd | Straight attenuated at the ends | Attenuated without calyptra | nd | | Cylindrical, shorter than wide | (8.13 ±0.92  width: length ratio 1.68 (± 0.65) | 5.48 (±2.01) | | Te et al. 2017 |

**Table S5.** Comparison of the morphological features of the *Laspinema* sp*.* PMC 878.14 strain with other *Laspinema* and *Phormidium* species belonging to the same phylogenetical cluster (Fig. 9). nd: not determined.

| **Taxonomic assignment** | **Strain** | **Origin** | **Habitat** | **Trichome** | | | | | | **Vegetative cell** | | | | | | | | | **Reference** |
| --- | --- | --- | --- | --- | --- | --- | --- | --- | --- | --- | --- | --- | --- | --- | --- | --- | --- | --- | --- |
|  |  |  |  | Color | Sheath | Shape | Shape of terminal cell | Width min.-max. (mean) µm | | | Shape | | Width min.-max. (mean), µm | | Length min.-max. (mean), µm | |  | | |
| *Laspinema* sp. | PMC 878.14 | France | Benthic on mud freshwater, brackish waters | Bright blue-green | Lacking sheath | Straight, nutated at the end | Obtuse-conical, rounded-conical, without calyptra | | 3.8 - 4.8 | | | Shorter than wide | | 3.8 - 4.8 (4.26 ±0.27) | | 2.3 - 3.3 (2.87 ±0.28) | | This study | |
| *Laspinema thermale* | HK S5 clone 2  (MF360989.1) | Iran, Mahallat | Thermal spring | Blue-green or olive green | Thin, inconspicuous | Straight in natural environment forming benthic mats, slightly constricted on cross walls | Rounded, conical, shortly bent | | 3 – 4 (5) | | | Shorter than wide | | 3 – 4 (5) | | 1 – 2,4 | | Heidari et al. 2018 | |
| *Laspinema lumbricale* | UTCC 476 (AF218375.1) | Antarctica | Saline pond on McMurdo Ice Shelf | nd | nd | nd | Rounded, straight | | nd | | | nd | | 5 – 6 (7) | | 2 - 4 | | Casamatta et al. 2005 | |
| *Laspinema etoshii* | KR2008/49 (KC014068.1) | Namibia Etosha National Park, Okandeka Spring | Hyposaline water puddles springs and wet soil | Pale bright blue- green | Hyaline, colorless | Solitary, straight  or curvy, not or slightly constricted | Round, conical;  straight or bent without calyptra | | nd | | | Mainly wider than long | | 5.5 ±1.5 | | 3.0 ±1.0 | | Dadheech et al. 2013 | |
| *Phormidium cf. okenii* | Led-Z (EU196644.1) | Czech Republ Lednice na Moravě park | a pond, benthos | Dark blue-green | Delicate colourless | Gradually narrowed towards end, distinctly constricted at cross-walls | Bent, elongated  usually rounded | | nd | | | Slightly shorter than wide or isodiametrical, sometimes longer than wide | | 5.6-6.5 (3.7-7.5 ; 6.1) cell length/width ratio 0.5-0.8 | | nd | | Lokmer 2007 | |
| *Phormidium cf. terebreformis* | KR2003/25 (AY575936.1) | Kenya, Hot spring, Lake Bogoria, | Hyposaline hot springs | nd | nd | Solitary, straight  to undulating Constricted | Rounded without calyptra | | nd | | | Mainly isodiametric | | 5.5 ±0.5 | | 3.0 ±1.0 | | Dadheech et al. 2013 | |

**Table S6.** Comparison of the morphological features of the *Microcoleus vaginatus* PMC 879.14 strain with other *Microcoleus* species or strains belonging to the same phylogenetical cluster (Fig. 9). nd: not determined.

| **Taxonomic assignment** | **Strain** | **Origin** | **Habitat** | **Trichome** | | | | | | **Vegetative cell** | | | | | | | | **Reference** | |  |  |
| --- | --- | --- | --- | --- | --- | --- | --- | --- | --- | --- | --- | --- | --- | --- | --- | --- | --- | --- | --- | --- | --- |
|  |  |  |  | Color | Sheath | Shape | Shape of terminal cell | Width min.-max. (mean) µm | | | Shape | | Width min.-max. (mean), µm | | Length min.-max. (mean), µm | |  | | | | |
| *Microcoleus vaginatus* | PMC 879.14 | France | Epilithic biofilm | Bright blue green | Thin, firm | Straight, cylindrical | Elongated, capitate,  with rounded  or truncated calyptra | | 4.7 - 6.2 | | | Shorter than wide | | 4.7 - 6.2 (5.63 ± 0,36) *5 (2014)* | | 2.4 - 4.8 (3.68 ± 0.52) *2 (2014)* | | | this study | |  |
| *Microcoleus autumnalis* | SAG 35.90 (EF654081.1) | Switzerland, brook in Verzascatal | Freshwater | nd | nd | nd | nd | | nd | | | nd | | nd | | nd | | | SAG site web collection | |  |
| *Microcoleus autumnalis* | Arct-Ph5 (DQ493873.2) | Canada, Ellesmere Island | Mineral soil | nd | Few filaments possessed | Single filaments or mucilaginous colonies | Rounded calyptra | | 8 – 9 | | | nd | | 3 – 4 | | 2 – 4 | | | Elster et al. 1997 | |  |
| *Microcoleus vaginatus* | CCALA 154 (JN230342.1) | Japan, East Asia | Cooling tower | nd | nd | nd | nd | | nd | | | nd | | nd | | nd | | | Strunecky et al. 2013 | |  |
| *Microcoleus vaginatus* | JR12 (JN230335.1) | Antarctica James Ross Island | Gray-black mats biofilm on rocks | nd | nd | nd | nd | | nd | | | nd | | nd | | nd | | | Strunecky et al. 2013 | |  |
| *Microcoleus vaginatus* | JR6 (JN230335.1) | Antarctica James Ross Island | Periphyton in wetlands close Lachman Lake | nd | nd | nd | nd | | nd | | | nd | | nd | | nd | | | Strunecky et al. 2013 | |  |
| *Microcoleus vaginatus* | A25 (JN230341.1) | Tennessee, North America | Soil | nd | nd | nd | nd | | nd | | | nd | | nd | | nd | | | Strunecky et al. 2013 | |  |
| *Oscillatoria amoena* | SAG 1459-7 (KM0119962.1) | Germany, Bot. Gard. Univ. Bonn | Freshwater | nd | nd | nd | nd | | nd | | | nd | | nd | | nd | | | SAG site web collection | |  |
| *Microcoleus vaginatus* |  | Cosmopolitan | Subaerophytic, on soils, stones, dried mud, waterfalls, littoral of lakes, rarely submersed in stagnant and flowing waters | Bright blue green or olive green to dirty green | Colourless, | Trichome long not constricted | Capitate, bluntly rounded, with conical, obtuse-conical or hemispherical calyptra | | (2,5) 3 - 7 (9?) | | | Shorter than wide | | 2 - 5 (6,7) | | nd | | | Komárek & Anagnostidis 2005 | |  |

**Table S7.** Comparison of the morphological features of the *Lyngbya martensiana* PMC 880.14 strain with other *Lyngbya martensiana* belonging to the same phylogenetical cluster (Fig. 9). nd: not determined.

| **Taxonomic assignment** | **Strain** | **Origin** | **Habitat** | **Trichome** | | | | | | **Vegetative cell** | | | | | | | | | **Reference** | |  |
| --- | --- | --- | --- | --- | --- | --- | --- | --- | --- | --- | --- | --- | --- | --- | --- | --- | --- | --- | --- | --- | --- |
|  |  |  |  | Color | Sheath | Shape | Shape of terminal cell | Width min.-max. (mean) µm | | | Shape | | Width min.-max. (mean), µm | | Length min.-max. (mean), µm | | |  | | | |
| *Lyngbya martensiana* | PMC 880.14 | France | Epilithic biofilm | Pale blue-green,  olive green | Thick, colourless | Cylindrical straight, flexuous | Widely rounded, hemispherical without calyptra | | 4.8 - 6.6 | | | Shorter than wide | | 4.8 - 6.6 (5.3 ± 0.47) | | 1.2 - 2.1 (1.6 ± 0.2) | This study | | |  |  |
| *Lyngbya martensiana* | AUS-JR/MT/NT-124 (KX670285.1) | India | Rice Field soil | Blue green | Colourless, firm, thick | Entangled and interwoven, long flexible, straight | Rounded without calyptra, not capitate | | 6-10 | | | Cells ½ -1/4 times long as broad | |  | | 1.5 – 2.5 | Thajamambi et al. 2016 | | |  |  |
| *Lyngbya martensiana* | MBDU 518 (KX913923.1) | India | Marine | nd | nd | nd | nd | | nd | | | nd | | nd | | nd | Genbank site web | | |  |  |
| *Lyngbya martensiana* | H3b/7 (JN854142.1) | Guatemala, Lake Atilan | Periphytic freshwater | Bright blue green | Firm, smooth from outside | Cylindrical, straight or slightly bent, coiled, or screw-like coiled | Not attenuated | | 9 | | | nd | | (6.2) 7 – 7.5 (8.7) | | 1.4 | Komárek et al. 2013  Turicchia et al. 2009 | | |  |  |

**Table S8.** Comparison of the morphological features of the *Nostoc* sp*.* PMC 881.14 strain with other *Nostoc* species belonging to the same phylogenetical cluster (Fig. 14). nd: not determined.

| **Taxonomic assignment** | | **Strain** | | **Origin** | | **Habitat** | **Colony morphology** | | | | **Vegetative cell** | | | **Akinete** | | | | | **Heterocyte** | | | **Reference** | |  |
| --- | --- | --- | --- | --- | --- | --- | --- | --- | --- | --- | --- | --- | --- | --- | --- | --- | --- | --- | --- | --- | --- | --- | --- | --- |
|  | |  | |  | |  | Sheath and shape | |  | Shape | | Width min.-max. (mean), µm | Length min.-max. (mean), µm | Shape | Width min.-max. (mean), µm | | Length min.-max. (mean), µm | Shape | | Width min.-max. (mean), µm | Length min.-max. (mean), µm | |  |  |
| *Nostoc* sp. | PMC 881.14 | | France | | Epilithic biofilm | | | nd |  | nd | | 2.8 – 5.5  (4.5) | 2.6 – 4.6 (3.4) | not observed | | nd | nd | +/- sub spherical | | 2.6 – 5,7 (3.4) | 2.3 – 5.1  (3.8) | | This study | |
| *Nostoc linckia* | IAM M-251 | | Japan | | nd | | | nd |  | nd | | nd | nd | nd | | nd | nd | nd | | nd | nd | | Genbank site web | |
| *Nostoc muscorum* | II | | Czech Republic | | Field | | | nd |  | nd | | nd | nd | nd | | nd | nd | nd | | nd | nd | | Rajaniemi et al 2005 | |
| *Nostoc ellipsosporum* | V | | Czech Republic | | Field | | | nd |  | nd | | nd | nd | nd | | nd | nd | nd | | nd | nd | | Rajaniemi et al 2005 | |
| *Nostoc edaphicum* | X | | Czech Republic | | Field (salty soils) | | | Spherical or oval sometimes irregular colourless or yellowish brown |  | Barrel shaped | | 3 - 4.2 | nd | not observed | |  |  | Mainly terminal, rarely intercalary | | nd | nd | | Singh et al 2020 | |
| *Nostoc calcicola* | III | | Czech Republic | | Field | | | nd |  | nd | | nd | nd | nd | | nd | nd | nd | | nd | nd | | Rajaniemi et al 2005 | |
| *Nostoc* sp. | 1tu14s8 | | Finland | | Lake | | | nd |  | nd | | nd | nd | nd | | nd | nd | nd | | nd | nd | | Rajaniemi et al 2005 | |
| *Nostoc* sp. | ATCC 53789 | | Scotland | | lichen thallus | | | nd |  | nd | | nd | nd | nd | | nd | nd | nd | | nd | nd | | Genbank site web | |
| *Nostoc punctiforme* | PCC 73102 | | Australia | | Root section, Marozamia sp. (Humid soil or cycad tubercules) | | | Dark blue green or blackish spherical sub aerophytic |  | Barrel shaped to spherical to ellipsoidal | | 2.6 – 5.5 (3 - 5) | nd | Present | | nd | nd | Barrel shaped to +/- sub spherical | | 4 -6.5 (diameter) |  | | Singh et al 2020 | |

**Table S9.** Comparison of the morphological features of the *Aliinostoc* sp*.* PMC 882.14 strain with other *Aliinostoc* and *Trichormus* species belonging to the same phylogenetical cluster (Fig. 14). no: not observed. nd: not determined.

|  |  |  |  | **Filament** | | | **Vegetative cell** | | | **Akinete** | | **Heterocyte** | | | | | **Reference** | |  |  |
| --- | --- | --- | --- | --- | --- | --- | --- | --- | --- | --- | --- | --- | --- | --- | --- | --- | --- | --- | --- | --- |
| **Taxonomic assignment** | **Strain** | **Origin** | **Habitat** | Sheath | Shape of terminal cell | Shape | Width min.-max. (mean), µm | Length min.-max. (mean), µm | Shape | Width min.-max. (mean), µm | Length min.-max. (mean), µm | Shape | Width min.-max. (mean), µm | | Length min.-max. (mean), µm | |  | | |  |
| *Aliinostoc* sp. | PMC 882.14 | France | Epilithic biofilm | nd | Conical | Spherical /cylindrical | 3.4 - 5.7 (4.5) | 2.7 - 6.5 (4.6) | oval | 4.6 - 7.4 (5.9) | 4.5 - 9.1 (7.2) | Spherical, intercalary | | 4.1 - 6.5 (5.4) | | 4.2 - 6.9 (5.4) | | This study | | |
| *Aliinostoc constrictum* | SA3O | Iran | Paddy field | thin hyaline | nd | spherical / cylindrical | 3.1 - 5.6 | 2.5 - 6.5 | no | nd | nd | spherical to oblong | | 3.0 - 6.8 | | 6.5 - 8.0 | | Kabirnataj et al. 2020 | | |
| *Aliinostoc soli* | ZH1(3) | India,  Pachmarhi | Soil dwelling | thin hyaline | Bluish green mats with leathery texture | Barrel shaped | 3.2 – 3.7 | 3.6 – 4.0 | Present | nd | nd | spherical | | 3.7 – 4.9 | | 3.8 – 4.3 | | Saraf et al. 2018 Kabirnataj et al. 2020 | | |
| *Aliinostoc tiwarii* | LI PS | India,  Mumbra | Freshwater | thin hyaline | Greenish blue mats with soft texture | Barrel shaped | 5.2 - 5.6 | 5.3 – 5.6 | Present | nd | nd | spherical | | 3.7 – 4.9 | | 3.8 – 4.3 | | Saraf et al. 2018 Kabirnataj et al. 2020 | | |
| *Trichormus dolium* | 1 | nd | nd | nd | Conical | nd | 1.6 - 3.0 (2.3) |  | lenticular | 1.9 - 3.7 (2.7) | 3.5 - 6.5 (4.6) |  | |  | |  | | Rajaniemi et al 2005 | | |
| *Trichormus variabilis* | KINDAK 2001/4 | Russia | Soil | nd | Conical | nd | 2.1 -9.6 (6.7) | nd | Oval, slightly compressed in the middle | 5 – 7.4 (6.9) | 8 – 14.8 (11.3) | nd | | nd | | nd | | Rajaniemi et al 2005 | | |
| *Trichormus azollae* | Kom-BAI | nd | nd | nd | Rounded | nd | 1.4 - 5.3 (4) | nd | oval | 7.5 - 7.5 (7.5) | 12.5 - 15 (14.5) | nd | | nd | | nd | | Rajaniemi et al 2005 | | |
| *Nostoc* sp. | PCC 7120 | nd | nd | nd | nd | nd | 3 - 3.5 |  | no | nd | nd | nd | | nd | | nd | |  | | |
| *Anabaena oscillarioides* | BO HINDAK 1984/43 | Canada | nd | nd | Conical | nd | 2.3 – 5.2 (3.8) | nd | no | nd | nd | nd | | nd | | nd | | Rajaniemi et al 2005 | | |
| *Minunostoc cylindricum* | CHAB 5843 | China | Small seven-hole Scenic area | blue green gelatinous | Cylindrical | nd | 2.5 - 3.8 (3.2) | nd | no | nd | nd | no | | nd | | nd | | Cai et al. 2019 | | |

**Table S10.** Comparison of the morphological features of the *Dulcicalothrix* sp*.* PMC 884.14 strain with other *Dulcicalothrix (*fka *Calothrix)* species belonging to the same phylogenetical cluster (Fig. 14). nd: not determined.

| **Taxonomic assignment** | **Strain** | **Origin** | **Habitat** | **Vegetative cell** | | | **Akinete** | **Heterocyte** | | | **Reference** |
| --- | --- | --- | --- | --- | --- | --- | --- | --- | --- | --- | --- |
|  |  |  |  | Shape | Width min.-max. (mean), µm | Length min.-max. (mean), µm | Shape | Shape | Width min.-max. (mean), µm | Length min.-max. (mean), µm |  |
| *Calothrix* sp. | PMC 884.14 | France | Epilithic biofilm | nd | 5.2 – 7.9  (6.6±0.7) | 4.8 – 6.2 (5.5±0.4) | not observed | hemispherical | 4.7-8.19 (6.2±0.9) | 3.48-7.33 (4.9±0.9) | This study |
| *Duclcalothrix necridiiformans* | V13 | India | Terrestrial, shallow water freshwater | wider near the basal heterocyte and continue to narrow towards the distal end | Distal cells:  3.63 – 3.69 Intercalary cells: 8.16 – 8.21 | Distal cells:  5.20 – 5.26 Intercalary cells: 6.20 – 6.26 | not observed | spherical sometimes slightly elongated end | 6.0 – 6.60 | 5.80 – 5.90 | Saraf et al  2019 |
| *Dulcicalothrix* *thermalis  (Calothrix* *thermalis)* | PCC 7715 | France | Thermal spring | Isodiametric to longer than wide cells | nd | nd | Present | nd | nd | nd | PCC site web collection |
| *Dulcicalothrix desertica (Calothrix desertica)* | PCC 7102 | Chile | Soil, fine desert sand | nd | nd | nd | nd | nd | nd | nd | PCC site web collection |
| *Dulcicalothrix* sp. *(Calothrix* sp.) | PCC 7103 | USA | Herbarium material of *Anacystis montana* | nd | nd | nd | nd | nd | nd | nd | PCC site web collection |
| *Dulcicalothrix* *parietina (Calothrix* *parietina)* | CCAP 1410/10 | England | Freshwater | nd | nd | nd | nd | nd | nd | nd | Genbank site web |

**Table S11.** Molecules selected for their antioxidant and/or anti-inflammatory properties (Demay et al. 2019) related to the genera of cyanobacteria isolated from Thermes de Balaruc-Les-Bains. MAAs: Mycosporine-like amino acids.

| **Targeted molecule families** | **Characteristics** | **Bioactivities** | **Genus producers** | **References** |
| --- | --- | --- | --- | --- |
| **Aeruginosins** | Peptide  Linear  NRPS  Hydrosoluble | No cytotoxicity  Anti-inflammatory activity  Protease inhibitor (trypsin, thrombin, plasmin) | ***Nostoc*** sp. Lukešová 30/93 | [1–4] |
| **Carotenoids** | Terpenoid  Pigment  Liposoluble | Antioxidant  Sunscreen | All cyanobacteria | [5–10] |
| **Chlorophylls** | Substituted tetra-pyrrole porphyrin molecule  Pigment  Liposoluble | Antioxidant  Antimutagenic  Chemopreventive  Photosensitizing act.  Cancer preventive agent | All cyanobacteria | [11–13] |
| **Honaucins** | Lactone  Linear  Liposoluble | Anti-inflammatory activity  No antioxydant act.  Quorum sensing inhibition | ***Leptolyngbya*** *crossbyana* HI09-1 | [14,15] |
| **Mycosporine-like amino acids (MAAs)** | Cyclohexenone-amino acid  Hydrosoluble | Antioxidant  UV-absorbing  UV-protective act.  Sunscreen  Antiphotoaging | ***Calothrix*** *parietina*  ***Calothrix*** sp.  ***Leptolyngbya*** sp.  ***Lyngbya*** *aestuarii*  ***Lyngbya*** sp. CU2555  ***Lyngbya*** sp.  ***Microcoleus*** *chthonoplastes*  ***Microcoleus*** *paludosus*  ***Microcoleus*** sp.  ***Nostoc*** *commune*  ***Nostoc*** *microscopicum*  ***Nostoc*** *punctiforme* ATCC 29133  ***Nostoc*** sp. | [5,16–25] |
| **Phycocyanins** | Protein  Pigment  Hydrosoluble | Antioxidant  Anti-inflammatory  Neuroprotective effects  Hepatoprotective effects | All cyanobacteria | [26–29] |
| **Phycoerythrins** | Protein  Pigment  Hydrosoluble | Antioxidant  Moderate aging | Several strains belonging to all orders | [30–32] |
| **Scytonemins** | Alkaloid  Pigment  Liposoluble | Anti-inflammatory  UV protection  Inhibit cell cycle kinases  Antiproliferative  Induction of autophagic death | ***Calothrix*** *crustacea*  ***Calothrix*** *parietina*  ***Calothrix*** sp.  ***Chroococcus*** sp.  ***Lyngbya*** *aestuarii*  ***Lyngbya*** sp.  ***Lyngbya*** sp. CU2555  ***Nostoc*** *commune* Vauch  ***Nostoc*** *microsopicum*  ***Nostoc*** *parmelioides*  ***Nostoc*** *pruniforme*  ***Nostoc*** *punctiforme* PCC 73102, ATCC 29133 | [16,33–38] |

**References Table S11**

1. Ishida, K.; et al. Aeruginosins, protease inhibitors from the cyanobacterium Microcystis aeruginosa. *Tetrahedron* **1999**, *55*, 10971–10988, doi:10.1016/S0040-4020(99)00621-3.

2. Matsuda, H.; et al. Aeruginosins 102-A and B, New Thrombin Inhibitors from the Cyanobacterium Microcystis viridis (NIES-102). *Tetrahedron* **1996**, *96*.

3. Fewer, D.P.; et al. New Structural Variants of Aeruginosin Produced by the Toxic Bloom Forming Cyanobacterium Nodularia spumigena. *PLoS One* **2013**, *8*, doi:10.1371/journal.pone.0073618.

4. Kapuścik, A.; et al. Novel aeruginosin-865 from Nostoc sp. as a potent anti-inflammatory agent. *ChemBioChem* **2013**, *14*, 2329–2337, doi:10.1002/cbic.201300246.

5. Choi, H.; et al. Honaucins A-C, potent inhibitors of inflammation and bacterial quorum sensing: Synthetic derivatives and structure-activity relationships. *Chem. Biol.* **2012**, *19*, 589–598, doi:10.1016/j.chembiol.2012.03.014.

6. Mascuch, S.J.; et al. Marine Natural Product Honaucin A Attenuates Inflammation by Activating the Nrf2-ARE Pathway. *J. Nat. Prod.* **2017**, *81*, 506–514, doi:10.1021/acs.jnatprod.7b00734.

7. Rastogi, R.P.; et al. Characterization of UV-screening compounds, mycosporine-like amino acids, and scytonemin in the cyanobacterium Lyngbya sp. CU2555. *FEMS Microbiol. Ecol.* **2014**, *87*, 244–256, doi:10.1111/1574-6941.12220.

8. Garcia-Pichel, F.; et al. Occurrence of UV-Absorbing, Mycosporine-Like Compounds among Cyanobacterial Isolates and an Estimate of Their Screening Capacity. *Appl. Environ. Microbiol.* **1993**, *59*, 163–9.

9. Shibata, K. Pigments and a UV-absorbing substance in corals and a blue-green alga living in the great barrier reef. *Plant Cell Physiol.* **1969**, *10*, 325–335, doi:10.1093/oxfordjournals.pcp.a074411.

10. Jain, S.; et al. Cyanobacteria as efficient producers of mycosporine-like amino acids. *J. Basic Microbiol.* 2017, *57*, 715–727.

11. Kageyama, H.; et al. *Mycosporine-Like Amino Acids as Multifunctional Secondary Metabolites in Cyanobacteria: From Biochemical to Application Aspects*; 1st ed.; Elsevier B.V., 2018; Vol. 59; ISBN 9780444641793.

12. Ryu, J.; et al. Protective effect of porphyra-334 on UVA-induced photoaging in human skin fibroblasts. *Int. J. Mol. Med.* **2014**, *34*, 796–803, doi:10.3892/ijmm.2014.1815.

13. Suh, H.-J.; et al. Mycosporine Glycine Protects Biological Systems Against Photodynamic Damage by Quenching Singlet Oxygen with a High Efficiency¶. *Photochem. Photobiol.* **2003**, *78*, 109, doi:10.1562/0031-8655(2003)078<0109:mgpbsa>2.0.co;2.

14. Matsui, K.; et al. Novel glycosylated mycosporine-like amino acids with radical scavenging activity from the cyanobacterium Nostoc commune. *J. Photochem. Photobiol. B Biol.* **2011**, *105*, 81–89, doi:10.1016/j.jphotobiol.2011.07.003.

15. Sakamoto, T.; et al. Four chemotypes of the terrestrial cyanobacterium Nostoc commune characterized by differences in the mycosporine-like amino acids. *Phycol. Res.* **2019**, *67*, 3–11, doi:10.1111/pre.12333.

16. Karsten, U.; et al. Carotenoids and mycosporine-like amino acid compounds in members of the Genus microcoleus (Cyanobacteria): A chemosystematic study. *Syst. Appl. Microbiol.* **1996**, *19*, 285–294, doi:10.1016/S0723-2020(96)80054-3.

17. Sinha, R.P.; et al. Database on mycosporines and mycosporine-like amino acids (MAAs) in fungi, cyanobacteria, macroalgae, phytoplankton and animals. *J. Photochem. Photobiol. B Biol.* **2007**, *89*, 29–35, doi:10.1016/j.jphotobiol.2007.07.006.

18. Stevenson, C.S.; et al. Scytonemin-a marine natural product inhibitor of kinases key in hyperproliferative inflammatory diseases. *Inflamm. res* **2002**, *51*, 112–114.

19. Stevenson, C.S.; et al. The identification and characterization of the marine natural product scytonemin as a novel antiproliferative pharmacophore. *J. Pharmacol. Exp. Ther.* **2002**, *303*, 858–866, doi:10.1124/jpet.102.036350.ever.

20. Itoh, T.; et al. Reduced scytonemin isolated from Nostoc commune induces autophagic cell death in human T-lymphoid cell line Jurkat cells. *Food Chem. Toxicol.* **2013**, *60*, 76–82, doi:10.1016/j.fct.2013.07.016.

21. Pathak, J.; et al. Genetic regulation of scytonemin and mycosporine-like amino acids (MAAs) biosynthesis in cyanobacteria. *Plant Gene* **2019**, *17*, 100172, doi:10.1016/J.PLGENE.2019.100172.

22. Fleming, E.D.; et al. Effects of nitrogen source on the synthesis of the UV-screening compound, scytonemin, in the cyanobacterium Nostoc punctiforme PCC 73102. *FEMS Microbiol. Ecol.* **2008**, *63*, 301–308, doi:10.1111/j.1574-6941.2007.00432.x.

23. Garcia-Pichel, F.; et al. Characterization and biological implications of scytonemin, a cyanobacterial sheath pigment. *J. Phycol.* **1991**, *27*, 395–409, doi:10.1111/j.0022-3646.1991.00395.x.

24. Stahl, W.; et al. Antioxidant activity of carotenoids. *Mol. Aspects Med.* 2003, *24*, 345–351.

25. Stahl, W.; et al. Bioactivity and protective effects of natural carotenoids. *Biochim. Biophys. Acta - Mol. Basis Dis.* **2005**, *1740*, 101–107, doi:10.1016/j.bbadis.2004.12.006.

26. Hirschberg, J.; et al. Carotenoids in Cyanobacteria BT - The Molecular Biology of Cyanobacteria. In *The Molecular Biology of Cyanobacteria*; Springer Netherlands: Dordrecht, 1994; pp. 559–579 ISBN 978-94-011-0227-8.

27. Joshi, D.; et al. Effect of UV-B Radiation and Desiccation Stress on Photoprotective Compounds Accumulation in Marine Leptolyngbya sp. *Appl. Biochem. Biotechnol.* **2018**, *184*, 35–47, doi:10.1007/s12010-017-2523-3.

28. Takaichi, S.; et al. Unique carotenoids in the terrestrial cyanobacterium nostoc commune nies-24: 2-hydroxymyxol 2??-fucoside, nostoxanthin and canthaxanthin. *Curr. Microbiol.* **2009**, *59*, 413–419, doi:10.1007/s00284-009-9453-4.

29. Lanfer-Marquez, U.M.; et al. Antioxidant activity of chlorophylls and their derivatives. In Proceedings of the Food Research International; Elsevier, 2005; Vol. 38, pp. 885–891.

30. Queiroz Zepka, L.; et al. Catabolism and bioactive properties of chlorophylls. *Curr. Opin. Food Sci.* **2019**, *26*, 94–100, doi:10.1016/j.cofs.2019.04.004.

31. Ferruzzi, M.G.; et al. Digestion, absorption, and cancer preventative activity of dietary chlorophyll derivatives. *Nutr. Res.* 2007, *27*, 1–12.

32. Romay, C.; et al. C-Phycocyanin: A Biliprotein with Antioxidant, Anti-Inflammatory and Neuroprotective Effects. *Curr. Protein Pept. Sci.* **2003**, *4*, 207–216, doi:10.2174/1389203033487216.

33. Remirez, D.; et al. Influence of C-phycocyanin on hepatocellular parameters related to liver oxidative stress and Kupffer cell functioning. *Inflamm. Res.* **2002**, *51*, 351–356, doi:10.1007/PL00000314.

34. Patel, A.; et al. Antioxidant potential of C-phycocyanin isolated from cyanobacterial species Lyngbya, Phormidium and Spirulina spp. *Indian J. Biochem. Biophys.* **2006**, *43*, 25–31, doi:10.1016/S0260-8774(98)00168-X.

35. Romay, C.; et al. Antioxidant and anti-inflammatory properties of C-phycocyanin from blue-green algae. *Inflamm. Res.* **1998**, *47*, 36–41, doi:10.1007/s000110050256.

36. Sonani, R.R.; et al. Concurrent purification and antioxidant activity of phycobiliproteins from Lyngbya sp. A09DM: An antioxidant and anti-aging potential of phycoerythrin in Caenorhabditis elegans. *Process Biochem.* **2014**, *49*, 1757–1766, doi:10.1016/j.procbio.2014.06.022.

37. Patel, S.N.; et al. Antioxidant activity and associated structural attributes of Halomicronema phycoerythrin. *Int. J. Biol. Macromol.* **2018**, *111*, 359–369, doi:10.1016/j.ijbiomac.2017.12.170.

38. Bryant, D.A. Phycoerythrocyanin and phycoerythrin: properties and occurrence in cyanobacteria. *J. Gen. Microbiol.* **1982**, *128*, 835–844, doi:10.1099/00221287-128-4-835.
