# Supplementary material for: Diversity of cyanobacteria from thermal muds (Balaruc-Les-Bains, France) with the description of Pseudochroococcus coutei gen. nov., sp. nov
Source: FEMS Microbes. 2021 Apr 22;2:xtab006. doi: 10.1093/femsmc/xtab006 (PMC10117791; doi:10.1093/femsmc/xtab006)
Supplement: xtab006_Supplemental_Files [file xtab006_supplemental_files.zip › Reviews and Responses for FEMSMC-2020-019.docx]

**Reviews for FEMSMC-2020-019**

*Reviewer 1: Anonymous*

For my assessment of this manuscript I divide the information given into three parts: (1) the isolation and description of a new genus and species of cyanobacteria; (2) the characterization of other known taxa from the environment investigated; (3) information about metabolites, natural compounds, therapeutic properties and genome sequences.

Part (1) is OK, and such a finding deserves publication. The authors did a good job here, see a few minor comments below.

Part (2) does not provide any significant new information beyond confirming that these other already known taxa are also found in the pond investigated and are very similar to other isolates reported earlier from other sites. Here it depends on the policy of the journal whether such confirmatory information can be published in spite of the fact that it adds very little news. Most journals I know would shorten the long pages of descriptions to a sentence such as “In addition to the new taxon described here, we obtained cultures of …, …. and … Table … summarizes their properties, and the 16S rRNA gene sequences and ITS sequences are available from GenBank under accession numbers …

Part (3) is fully irrelevant. Although it was stated that genomic, metabolomics and bioactivities studies were performed (p. 5, line 4), no results were given. Comments such as given in the first and last lines of the Abstract and the first paragraph of the Introduction are out of place. If the study is on taxonomy, the authors must refrain from mentioning other aspects irrelevant to the paper. The paragraph on ‘potential therapeutic properties’ on p. 16 must be deleted as nothing here is supported by the results presented. Such speculations have no place in a scientific publication. And the first paragraph of p. 15 is irrelevant as well as the authors did not determine and compare genome sequences of their isolates.

Minor comments:

Graphical abstract: Cyanobacterium, not Cyanobacteria. What is the meaning of the r after couteii?

About the proposed generic name: as far as I could ascertain, the ICN allows hyphens in generic name (Article 20.3). Still, in view of the many cases of cyanobacterial compound generic names in which Pseudo is not followed by a hyphen, I suggest Pseudochroococcus instead of Pseudo-chroococcus.

About the specific epithet couteii: If I understand Article 60.8 (a) of the ICN well, couteii is not correct, and must be corrected to coutei.

I understand that the basin from which the strains were isolated is the outflow of a hot spring. But except for the fact that cultures were incubated at 25 oC I did not find any information about the temperatures to which the organisms were exposed in situ. I am also missing information about the upper temperature limits at which the organisms could be grown.

p. 3, line 37: Is 5 g/l agar enough to give a solid medium? I doubt it.

References section: needs some work as the style used is not consistent. Do not capitalize words in article titles such as was done e.g. in the references Cuzman et al. 2010, Demay et al. 2019. Check the required style of the journal. Reference Hamlaoui 2014: where was this published? Reference Parveen et al. 2013: change n/a—n/a to the real volume and page numbers. These are examples only, there may be more.

*Reviewer 2: Anonymous*

The MS is overall well-structured and ticks in positively almost all the columns of a good taxonomic piece of work especially in context of modern cyanobacterial systematics.

But, there are few problems which need to be addressed more effectively. I will detail them below:

1. I do not understand that what purpose the Figs 4 and 5 serve separately. In a typical strong phylogeny, one can have simply one 16S rRNA gene based tree with usually 3 algorithms. Why do the authors need to complicate this by adding an ITS tree (which is not exactly very informative also)? Please think of it.

2. The 16S rRNA gene phylogeny is good but there is a bit of confusion. What exactly is the cluster of the new genus Pseudo-chroococcus? I am little perplexed. Is it a single species genus? If yes, then please be aware that it could be a big problem. This is not exactly the best way to describe a new genus. People have done it before but in many cases they had strong clusters and not just a single sequence. Do the authors have stronger evidence? How do they intend to treat the neighbouring OTUs Cyanobacteria sp. YW-2019b etc and Chroococcus-like CAWBG110 etc. They need to come clear on this before this new genus could be created.

3. At many places the authors depend a bit too much on percentage similarity values and cut-offs. This may not be the best way to dwell into cyanobacterial systematics. These cut-off values are better maybe in bacterial taxonomy but in cyanobacterial taxonomy, it will be much better and clearer if strong monophyletic groups having multiple members are created. In this work, it is not evident.

4. The authors have perhaps done direct sequencing of the amplicons and that is why only the D1-D1’ conserved region could be sequenced. Am I correct? If yes then the new genus could further be in a bit of tight position as the boxB and V3 regions should have been here. I know that this may not happen now, but please do think of it seriously.

5. The Calothrix phylogeny needs to be re-done. Strangely, the authors missed the work of Saraf et al (2019) where the family Calotrichaceae was re-established and the genus Dulcicalothrix was erected. The Calothrix here needs to be re-run and maybe it could fall into the Dulcicalothrix cluster. Please do this phylogeny again. This will change the entire treatment of Calothrix in the present work.

6. Why does the conclusion focus so much on WGS? It will be better if the authors conclude with their own results which in itself are quite good (though the interpretations are not satisfactory still). Whether WGS is good or not and how effective it will be in the future is still a matter of debate. As of now, I personally do not find the WGS way of systematics very encouraging. Cyanobacteria are special organisms, have beautiful morphologies, unprecedented heterogeneity and complex life cycles. It is doubtful whether the WGS way of taxonomy can address these intricacies in a holistic way.

7. The work overall is good, has been written nicely and well explained. But in spite of all this, suffers deeply in many aspects that I have mentioned above. It will be much better if this could be revised. I recommend a MAJOR REVISION.

*Author Response:*

FEMS Microbes
Reviewers' Comments to Authors:

Reviewer: 1
Comments to the Author
For my assessment of this manuscript I divide the information given into three parts: (1) the isolation and description of a new genus and species of cyanobacteria; (2) the characterization of other known taxa from the environment investigated; (3) information about metabolites, natural compounds, therapeutic properties and genome sequences.
Part (1) is OK, and such a finding deserves publication. The authors did a good job here, see a few minor comments below.

Response:
Many thanks for your comments. The revision following the minor comments have been done.

Part (2) does not provide any significant new information beyond confirming that these other already known taxa are also found in the pond investigated and are very similar to other isolates reported earlier from other sites. Here it depends on the policy of the journal whether such confirmatory information can be published in spite of the fact that it adds very little news. Most journals I know would shorten the long pages of descriptions to a sentence such as “In addition to the new taxon described here, we obtained cultures of …, …. and … Table … summarizes their properties, and the 16S rRNA gene sequences and ITS sequences are available from GenBank under accession numbers …

Response:
The thermal mud of Balaruc-les-Bains represents a particular environment. Baudinat (1986) and Dupuis (1987) initially identified a dominance of cyanobacteria belonging to the genera Phormidium and Oscillatoria, then in a second step a dominance of Phormidium africanum, Phormidium autumnale, Lyngbya sp., Chroococcus species minor and Anabaena sp. (Baudinat 1986; Dupuis 1987). Morphologically, these genera and/or species have morphological characteristics identical to the cyanobacteria studied in this ms, with, for example, the species Pseudochroococcus coutei PMC 885.14 which presents the same morphological criteria than the genus Chroococcus. However, the sequence of the 16S rRNA shows an affiliation clearly different from the Chroococcus cyanobacteria which led us to the description of a new genus and species.
In this ms, we describe the nine isolated strains belonging to four orders. As it is known, the phylogenies and taxonomy of cyanobacteria are changing and the genera belonging to the Oscillatoriales are in permanent revision. It is therefore important to present the results in a detailed way to describe the taxon.
As recommended and in order to lighten the ms, some figs have been moved to supp data (submitted version: Fig. 5, 8, 9 and 14).

Part (3) is fully irrelevant. Although it was stated that genomic, metabolomics and bioactivities studies were performed (p. 3, line 4), no results were given. Comments such as given in the first and last lines of the Abstract and the first paragraph of the Introduction are out of place. If the study is on taxonomy, the authors must refrain from mentioning other aspects irrelevant to the paper. The paragraph on ‘potential therapeutic properties’ on p. 16 must be deleted as nothing here is supported by the results presented. Such speculations have no place in a scientific publication. And the first paragraph of p. 15 is irrelevant as well as the authors did not determine and compare genome sequences of their isolates.

Response:
Concerning the first comment “Although it was stated that genomic, metabolomics and bioactivities studies were performed (p. 3, line 4), no results were given” we agree that no results are presented in this ms.
Our objective in this ms is to describe the nine strains and to correctly affiliate them before further studies. Based on this taxonomic affiliation, we were able to publish the results on their potential beneficial activities (Demay et al, 2021, Biomolecules doi.org/10.3390/biom11010028). The main results published in Demay et al, 2021 concern the production of antioxidant and/or anti-inflammatory compounds using genomic, metabolomic, and bioactivity analyses.
We considered that a full and correct taxonomic description is a pre-requisite for further studies, using the standard preconized to date for cyanobacteria.
However, as recommended by the reviewer, we modified the text to refrain some aspects.

Minor comments:
Graphical abstract: Cyanobacterium, not Cyanobacteria. What is the meaning of the r after couteii?

Response:
Many thanks for your comment concerning cyanobacterium. We have changed in the graphical abstract. We don’t see the “r” after coutei.

About the proposed generic name: as far as I could ascertain, the ICN allows hyphens in generic name (Article 20.3). Still, in view of the many cases of cyanobacterial compound generic names in which Pseudo is not followed by a hyphen, I suggest Pseudochroococcus instead of Pseudo-chroococcus.

Response:
Many thanks for this comment and we modified Pseudo-chroococcus in Pseudochroococcus.

About the specific epithet couteii: If I understand Article 60.8 (a) of the ICN well, couteii is not correct, and must be corrected to coutei.

Response:
Thank you for this attentive comment and recommendation; we modified “couteii” in “coutei”.

I understand that the basin from which the strains were isolated is the outflow of a hot spring. But except for the fact that cultures were incubated at 25oC I did not find any information about the temperatures to which the organisms were exposed in situ. I am also missing information about the upper temperature limits at which the organisms could be grown.

Response: The thermal mud of Balaruc Les Bains represents a very specific environment with a specific ion composition (e.g. Cl- 6.8 g.L-1 ; Na+ 3.7 g.L-1 ; SO42- 0.9 g.L-1). The Balaruc thermal waters gush out of the ground at a temperature of about 50°C. This composition cannot be assigned to hot spring. Concerning the in situ temperature (mimicking the historical maturation of the peloid in the open air), it varies from 0 to 30°C, depending of the season. However, during the sampling campaign (from April to October 2014), the average water temperature in the maturation basin was 27.3°C.
Concerning the upper temperature limits for growth, experimentations in laboratory have to be done to test these limits, for each strain.

p. 3, line 37: Is 5 g/l agar enough to give a solid medium? I doubt it.

Response:
We use the semi-solid medium with 5.L-1of agar to allow migration of motile filaments. This method is described in Rippka (1988).

References section: needs some work as the style used is not consistent. Do not capitalize words in article titles such as was done e.g. in the references Cuzman et al. 2010, Demay et al. 2019. Check the required style of the journal. Reference Hamlaoui 2014: where was this published? Reference Parveen et al. 2013: change n/a—n/a to the real volume and page numbers. These are examples only, there may be more.

Response:
Many thanks for your comments. Hamlaoui 2014 has been moved and replaced in (unpublished data). The revision following the FEMS Microbes recommendations and style have been done.

Reviewer: 2
Comments to the Author
The MS is overall well-structured and ticks in positively almost all the columns of a good taxonomic piece of work especially in context of modern cyanobacterial systematics.
But, there are few problems which need to be addressed more effectively. I will detail them below:
1. I do not understand that what purpose the Figs 4 and 5 serve separately. In a typical strong phylogeny, one can have simply one 16S rRNA gene based tree with usually 3 algorithms. Why do the authors need to complicate this by adding an ITS tree (which is not exactly very informative also)? Please think of it.

Response:
Many thanks for this comment. The ITS tree is based on sequences with more informative site (1915 nt) instead of the 16S rRNA sequences (1560 nt). The use of ITS plus 16S rRNA sequences allow to confirm the phylogenetic position of P. coutei.
We follow your recommendation and as the 16S rRNA gene-based tree is informative enough for the ms, the ITS tree has been moved in supp data materials (Fig S3).

2. The 16S rRNA gene phylogeny is good but there is a bit of confusion. What exactly is the cluster of the new genus Pseudo-chroococcus? I am little perplexed. Is it a single species genus? If yes, then please be aware that it could be a big problem. This is not exactly the best way to describe a new genus. People have done it before but in many cases they had strong clusters and not just a single sequence. Do the authors have stronger evidence? How do they intend to treat the neighboring OTUs Cyanobacteria sp. YW-2019b etc and Chroococcus-like CAWBG110 etc. They need to come clear on this before this new genus could be created.

Response:
As shown in Fig. 4 the P. coutei strain PMC 885.14 forms a clade with the 4 Cyanobacteria sp. YW-2019b clones. Our results show that this cluster is clearly distinct from the Chroococcus cluster. Based on the rule that a taxonomic unit must be included in only one phylogenetic lineage (Komárek, 2018) we therefore propose that all the strains from this cluster will be renamed with the new genus Pseudochroococcus. Furthermore, the 16SrRNA sequences identity of these strains were < 95% with others Chroococcus sequences.
To confirm this result, polyphasic approaches (morphological and ultrastructural) have to be done to confirm or not the species differences.

Concerning the Chroococcus-like CAWBG, the four strains showed a percentage of identity < 95% and forms a new genus. Furthermore, they were isolated from a marine environment (Wood et al., 2017). Then, further studies with polyphasic approaches are needed to better assign these cyanobacteria.

3. At many places the authors depend a bit too much on percentage similarity values and cut-offs. This may not be the best way to dwell into cyanobacterial systematics. These cut-off values are better maybe in bacterial taxonomy but in cyanobacterial taxonomy, it will be much better and clearer if strong monophyletic groups having multiple members are created. In this work, it is not evident.

Response:
We agree with this comment, the cut-off values for genus definition remain unclear in several publications. In this work we used a cut-off of 95% of 16S rRNA gene sequences identity for genus definition as proposed by Komárek (2010) (cf Materials and methods). We also confirm the phylogeny origins of the studied strain with 16S RNA trees with a well assigned cyanobacteria available in GenBank with references and the genus affiliation was based on the rule that a taxonomic unit must be included in only one phylogenetic lineage (Komárek, 2018).
For P. coutei, there is a clear cluster with 4 strains named Cyanobacteria sp. YW-2019b and we considered this cluster strong enough with 5 different members for a new genus assignment.

4. The authors have perhaps done direct sequencing of the amplicons and that is why only the D1-D1’ conserved region could be sequenced. Am I correct? If yes then the new genus could further be in a bit of tight position as the boxB and V3 regions should have been here. I know that this may not happen now, but please do think of it seriously.

Response.
Yes, this comment is correct. We only sequenced the D1-D1’ conserved region because the full ITS region was not available either after amplification nor considering the whole genome sequences. Some nucleotides were missing. But, fortunately, we amplified and we confirmed by WGS the same region than other genus close to Chroococcus (see Fig 6, Inacoccus, Cryptococcum, Chroococcus). We were able to then compare this region and to clearly show the differences between the genera.
Within the Pseudochroococcus, the organization of the D1-D1’ conserved region of P. coutei shows some differences with the Cyanobacteria sp. YW-2019b strains. This result confirms one genus for the 4 strains within this cluster with two different species.

5. The Calothrix phylogeny needs to be re-done. Strangely, the authors missed the work of Saraf et al (2019) where the family Calotrichaceae was re-established and the genus Dulcicalothrix was erected. The Calothrix here needs to be re-run and maybe it could fall into the Dulcicalothrix cluster. Please do this phylogeny again. This will change the entire treatment of Calothrix in the present work.

Response:
Many thanks for this comment and for this reference. We are sorry, we had missed this one. The Calothrix phylogeny has been re-done and the strain PMC 884.14 clustered with the Dulcicalothrix type strain. Then, we renamed Calothrix in Dulcicalothrix in all the ms (text, Figs, Table). The reference Saraf et al (2019) has been added.

6. Why does the conclusion focus so much on WGS? It will be better if the authors conclude with their own results which in itself are quite good (though the interpretations are not satisfactory still). Whether WGS is good or not and how effective it will be in the future is still a matter of debate. As of now, I personally do not find the WGS way of systematics very encouraging. Cyanobacteria are special organisms, have beautiful morphologies, unprecedented heterogeneity and complex life cycles. It is doubtful whether the WGS way of taxonomy can address these intricacies in a holistic way.

Response:
We agree with this comment and genome sequencing alone will not solve the taxonomy of cyanobacteria. On the other hand, several scientists are interested in cyanobacteria, such as chemists and biochemists for the properties of cyanobacteria and their metabolites. To date, genome sequencing is a simple, rapid tool that is becoming more and more used by this scientific community. We believe that with these data, becoming more and more available, we will have access to sequences data (16S and/or ITS) that can feed into cyanobacterial phylogenies. To date, we are convinced that our study shows that the polyphasic approach remains the most appropriate. One of the constraints also remains that of having strains in culture or in collections, for this type of study.

7. The work overall is good, has been written nicely and well explained. But in spite of all this, suffers deeply in many aspects that I have mentioned above. It will be much better if this could be revised. I recommend a MAJOR REVISION.

Response:
We hope that the modifications that we have done as suggested by the reviewers improve the manuscript for publication in FEMS Microbes.

**Reviews for FEMSMC-2020-019.R1**

No comments to authors

*Author response:*

Dear Editor,

First of all, many thanks to have considered the revised version of our manuscript submitted to FEMS.
As you suggested, please find a new more cohesive title:"Diversity of cyanobacteria from thermal muds (Balaruc-Les-Bains, France) with the description of Pseudochroococcus coutei (gen. & sp. nov.)"
We hope this modification improve the manuscript for publication in FEMS Microbes.

**Reviews for FEMSMC-2020-019.R2**

*Reviewer 1: Anonymous*

Except for the small correction in the title (... coutei gen. nov., sp. nov.) nomenclature aspects look OK (under the rules of the ICN).
